# TWEAK/Fn14 Activation Participates in Ro52-Mediated Photosensitization in Cutaneous Lupus Erythematosus

**DOI:** 10.3389/fimmu.2017.00651

**Published:** 2017-05-31

**Authors:** Yale Liu, Meifeng Xu, Xiaoyun Min, Kunyi Wu, Ting Zhang, Ke Li, Shengxiang Xiao, Yumin Xia

**Affiliations:** ^1^Department of Dermatology, The Second Affiliated Hospital, School of Medicine, Xi’an Jiaotong University, Xi’an, China; ^2^Core Research Laboratory, The Second Affiliated Hospital, School of Medicine, Xi’an Jiaotong University, Xi’an, China

**Keywords:** TWEAK, Fn14, Ro52, ultraviolet B, tumor necrosis factor receptor, keratinocyte, photosensitization, cutaneous lupus erythematosus

## Abstract

Tumor necrosis factor (TNF)-like weak inducer of apoptosis (TWEAK) binds to its sole receptor fibroblast growth factor-inducible 14 (Fn14), participating in various inflammatory responses. Recently, TWEAK/Fn14 activation was found prominent in the lesions of cutaneous lupus erythematosus (CLE). This study was designed to further reveal the potential role of this pathway in Ro52-mediated photosensitization. TWEAK, Fn14, and Ro52 were determined in the skin lesions of patients with CLE. Murine keratinocytes received ultraviolet B (UVB) irradiation or plus TWEAK stimulation and underwent detection for Ro52 and proinflammatory cytokines. The chemotaxis of J774.2 macrophages was evaluated on TWEAK stimulation of cocultured keratinocytes. We found that TWEAK, Fn14, and downstream cytokines were highly expressed in CLE lesions that overexpressed Ro52. Moreover, TWEAK enhanced the UVB-induced Ro52 upregulation in murine keratinocytes. Meanwhile, TWEAK stimulation of keratinocytes favored the migration of macrophages through promoting the production of chemokine C–C motif ligands 17 and 22. Furthermore, Fn14 siRNA transfection or nuclear factor-kappa B (NF-κB) inhibitor abrogated the TWEAK enhancement of Ro52 expression in keratinocytes. Similarly, TNF receptor associated factor 2 (TRAF2) siRNA reduced the protein level of Ro52 in these cells upon TWEAK stimulation. Interestingly, UVB irradiation increased the expression of TNF receptor type 1 (TNFR1) but not affecting TNFR2 expression in keratinocytes. In conclusion, the TWEAK/Fn14 signaling participates in Ro52-mediated photosensitization and involves the activation of NF-κB pathway as well as the function of the TRAF2/TNFR partners.

## Introduction

Although the incidence of cutaneous lupus erythematosus (CLE) has rarely been investigated, cutaneous manifestations appear in 72–85% of patients with systemic lupus erythematosus and represent the first sign of the disease in 23–28% of patients (1). CLE may appear in any area although it is frequently found in sun-exposed skin. Histologically, CLE is characterized by epidermal atrophy, apoptotic keratinocytes, and infiltration of inflammatory cells due to accumulated proinflammatory cytokines (2). In fact, CLE is not only indicative of cutaneous inflammation but also associated with systemic lupus disease activity and even psychiatric disease (3, 4). Epidemiologic studies have reported that up to 23% of patients with CLE develop systemic lupus erythematosus, with some progressing over several years (5). To elucidate the inflammatory pathogenesis in CLE is thus desirable for both monitoring and detaining the progression of such disease.

As one of the most characteristic symptoms of CLE, photosensitivity is prominent during the early onset or acute stage of the disease. Currently, photosensitivity in CLE is believed to be a consequence of ultraviolet irradiation based on genetic background (6). Ultraviolet B (UVB) irradiation upregulates Ro52 in cutaneous keratinocytes (7), which may further trigger the production of autoantibodies to Ro52 (8). Ro52 is an E3 ubiquitin ligase with a regulatory role in local inflammation, especially in photo-provoked skin (7). Through the ubiquitination of transcription factors, Ro52 regulates the production of type 1 interferon and other cytokines (9). Moreover, anti-Ro52 antibodies accumulate in CLE skin and also exacerbate local inflammatory responses. Therefore, Ro52-mediated photosensitization is central in the pathogenesis of CLE.

Tumor necrosis factor (TNF)-like weak inducer of apoptosis (TWEAK) is a mediator of proinflammatory factors. TWEAK engages its sole receptor fibroblast growth factor-inducible 14 (Fn14) and modulates various cell types under inflammatory condition (10–12). TWEAK/Fn14 interaction plays an important role in the pathogenesis of tissue injuries in systemic lupus erythematosus, including lupus nephritis (13), neuropsychiatric disease (14), and cardiovascular disease (15). Besides, the TWEAK/Fn14 pathway is activated in skin lesions of MRL/lpr lupus-prone mice (16), and Fn14 deficiency protects these mice from CLE inflammation induced by UVB irradiation (17). UVB irradiation promotes Fn14 expression, whereas TWEAK upregulates the production of regulated on activation normal T cell expressed and secreted (RANTES) and induces the apoptosis of keratinocytes (16). Thus, TWEAK/Fn14 activation contributes to the development of CLE in murine lupus model and is assumed to function similarly in patients with CLE.

Considering the facts that both Fn14 and Ro52 are upregulated in keratinocytes on UVB irradiation, we speculated an intrinsic connection between the TWEAK/Fn14 signals and Ro52 expression in skin. However, the effect of TWEAK/Fn14 activation on Ro52-mediated photosensitization has not been studied thus far. Therefore, this study was designed to investigate the potential role of the TWEAK/Fn14 pathway in the pathogenesis of photo-provoked injury in CLE.

## Materials and Methods

### Tissue Collection

Skin biopsies (lesional and non-lesional) were collected from patients (*n* = 15) who had been diagnosed according to CLE criteria (1) and received neither pharmaceutical nor physical therapy. Normal skin tissues (*n* = 10) were obtained from healthy donors undergoing plastic surgeries. The demographic characteristics of CLE patients and healthy donors were detailed in Table S1 in Supplementary Material. Each biopsy was processed routinely for both paraffin sections and fresh tissue lysates, the latter of which were used for total RNA extraction. This study was carried out in accordance with the recommendations of the guidelines of the Hospital Research Ethics Committee. All subjects gave written informed consent in accordance with the Declaration of Helsinki.

As described previously (17), female MRL/lpr mice (or MRL/MpJ mice) at 10 weeks of age were irradiated with UVB at a dose of 50 mJ/cm^2^. Irradiation was performed on the flank daily and lasted 2 days. There were five mice in each group. Skin biopsies were collected 24 h after last irradiation. The protocol was approved by the Research Ethics Committee.

### Immunohistochemistry

As described previously (18), paraffin sections were deparaffinized and rehydrated, followed by the addition of dual endogenous enzyme block (DAKO, Glostrup, Denmark). Rabbit anti-Fn14 (clone #EPR3179) or RANTES (catalog #ab9679) IgG (Abcam, Cambridge, MA, USA) and rabbit anti-TWEAK (catalog #sc-5558) or Ro52 (catalog #sc-20960) IgG (Santa Cruz, Dallas, TX, USA) were used as primary antibodies (4 µg/ml, overnight at 4°C). To exclude the possibility of non-specific staining, rabbit polyclonal IgG (catalog #A7016; Beyotime Biotech, Beijing, China) was used as control. Then, the sections were incubated with polymer-horseradish peroxidase-labeled goat anti-rabbit/mouse IgG (catalog #K4065) and 3,3'-diaminobenzidine-chromogen substrate (DAKO) in order. Finally, sections were counterstained with hematoxylin–eosin solution.

The positive staining areas were measured by ImageJ1.61u software (National Institutes of Health, Bethesda, MD, USA) (19). Six to eight viewing fields were randomly selected from epidermal areas within each section. Positive staining areas were expressed as a percentage of the whole field area (% positivity per square millimeter). Such quantitative analysis was performed by two pathologists in a blinded fashion.

### Cell Culture

Murine keratinocytes (PAM212 cell line) were cultured in Eagle’s minimal essential media supplemented with 10% fetal bovine serum, 4-(2-hydroxyethyl)-1-piperazineethanesulfonic acid buffer, l-glutamine, MEM non-essential amino acids, sodium pyruvate, and penicillin/streptomycin (Gibco, Waltham, MA, USA). Before the stimulation assays, keratinocytes were starved in 2% fetal bovine serum-supplemented medium for 24 h. UVB irradiation was done by using a 302-nm UVB instrument (Rayminder, Ottawa Hills, OH, USA). The UVB dose (5.5 mJ/cm^2^) was chosen based on preliminary experiments (Figure S1 in Supplementary Material). In some experiments, culture media were added with recombinant mouse TWEAK (100 ng/ml, 2 days; R&D Systems, Minneapolis, MN, USA) or bovine serum albumin (BSA; 100 ng/ml, 2 days; Sigma-Aldrich, St. Louis, MO, USA) immediately after UVB irradiation. The inhibitors of JSH-23 (20 µM; Sigma-Aldrich), U0126 (10 µM; Cell signaling, Danvers, MA, USA), and wortmannin (25 µg/ml; Beyotime Biotech) were added 2 h before the UVB and TWEAK treatments.

### Quantitative Reverse Transcription Polymerase Chain Reaction (qRT-PCR)

Total RNA was extracted from fresh tissues or cell cultures by using the PureLink RNA kit (Invitrogen, Grand Island, NY, USA). A commercial cDNA kit (Applied Biosystems, Carlsbad, CA, USA) was used for reverse transcription. qRT-PCR was carried out on the 7900HT Fast PCR system (Applied Biosystems), with SYBR Green Master Mixes (Invitrogen, Grand Island, NY, USA) as fluorescent dye. The expression levels of the objective genes were calculated by using the 2^−ΔCt^ method (20). The sequences of the primers (AuGCT DNA-SYN Biotech, Beijing, China) are shown in Table S2 in Supplementary Material.

### siRNA Transfection

siRNA transfection was done as described previously (11). In brief, keratinocytes reaching 50% confluence were incubated with a mixture of target (or control) siRNA and Lipofectamine 2000 reagent (Life Technologies, Carlsbad, CA, USA) for 24 h. The sequences of Fn14 siRNA were CCCAUACUAAGGAACUGCATT (sense) and UGCAGUUCCUUAGUAUGGGTC (antisense). The sequences of TNF receptor associated factor 2 (TRAF2) siRNA were GGUACACUAUGAGGUCUGCTT (sense) and GCAGACCUCAUAGUGUACCTC (antisense). The sequences of Ro52 siRNA were GCCUAUGAGUAUCGAAUGUTT (sense) and ACAUUCGAUACUCAUAGGCTC (antisense). The target genes were verified by qRT-PCR in total RNA extractions, showing an efficiency of >80%.

### Western Blotting

Cell cultures were extracted for protein lysates. After electrophoresis and transfer onto membranes, the samples were incubated with rabbit anti-Ro52 IgG (2 µg/ml; Abcam). Similarly, rabbit anti-TNF receptor type 1 (TNFR1; catalog #13377), TNFR2 (catalog #3727), cellular inhibitor of apoptosis protein 1 (cIAP1; catalo #4952), TNF receptor-associated factor 2 (TRAF2; catalog #4712), extracellular signal-regulated kinase (ERK; catalog #4695), p-ERK (catalog #4370), Akt (catalog #9272), or p-Akt (catalog #5012) IgG (Cell signaling) and rabbit anti-nuclear factor-kappa B (NF-κB) p50 (clone #E381), NF-κB p65 (catalog #ab16502), or β-actin (clone #SP124) (Abcam) were used as primary antibodies (2 µg/ml). Next, biotinylated goat anti-rabbit IgG (catalog #A0277; 2 µg/ml; Beyotime Biotech) was added. Horseradish peroxidase-streptavidin and an ECL kit (Thermo Scientific, Waltham, MA, USA) were used for color development. The ImageJ1.61u software was used for quantitation of the band intensities, which were then normalized to the β-actin values accordingly.

### Flow Cytometry and Immunofluorescence

Flow cytometry was carried out by using phycoerythrin-conjugated IgG targeting TNFR1 (catalog #sc-12746) or TNFR2 (catalog #sc-12750) (Santa Cruz). In brief, keratinocytes were digested with 0.25% trypsin–EDTA solution and then collected from dishes. Cells were blocked with 2% BSA in phosphate buffer saline for 1 h at 4°C, followed by twice washing. Primary antibodies (2 µg/ml) were added for 2 h at 4°C. After washing again, the stained cells were detected by using an LSRII instrument (BD Biosciences, San Jose, CA, USA). All data were analyzed with the FlowJo 7.6.1 software (Tree Star, Ashland, OR, USA).

Apoptotic cells were detected by flow cytometry (16). Keratinocytes were transfected with control or Ro52 siRNA, followed by UVB irradiation, TWEAK stimulation, or their combination. Cells were harvested and resuspended in annexin binding buffer. Annexin-phycoerythrin and 7-aminoactinomycin D (BD Biosciences) were added to samples in order. Flow cytometry was performed immediately. In this assay, early apoptotic cells were annexin positive but 7-aminoactinomycin D negative, while late apoptotic cells were positive for both of them.

Immunofluorescence was performed as reported previously (21). Briefly, keratinocytes were grown on a glass-bottom culture dish (MatTek, MA, USA), and incubated with fluorescein isothiocyanate-labeled antibodies targeting Ro52 (clone #sc-25351), TNFR1 (clone #sc-12746), or TNFR2 (clone #sc-12750) (2 µg/ml; Santa Cruz). After routine washing, cells were observed under a digital confocal microscopy (Leica Co., Wetzlar, Germany). Immunofluorescence was also carried out with frozen sections. Goat anti-Ro52 IgG (catalog #sc-21362; Santa Cruz) and rabbit anti-Fn14 IgG (clone #EPR3179; Abcam) were primary antibodies (2 µg/ml). The Alexa 488-conjugated donkey anti-goat IgG (catalog #ab150129) and Alexa 594-conjugated donkey anti-rabbit IgG (catalog #ab150076) were used as secondary antibodies (2 µg/ml; Abcam). Finally, sections were counterstained with 4′,6-diamidino-2-phenylindole before observation under digital confocal microscopy.

### Macrophage Chemotaxis

*In vitro* chemotaxis of macrophages was determined as described previously (18), but with some modifications. Briefly, keratinocytes were grown in six-well plates and stimulated with TWEAK or BSA (10 µg/ml, 2 days). Murine macrophages (J774.2 clone, 1 × 10^6^/ml) were transferred to an inner insert with an 8-µm pore membrane (Corning Inc., Corning, NY, USA), which allowed macrophage migration. The inner insert was placed in an outer insert (pore size = 0.4 µm) that selectively blocked the penetration of TWEAK (27.22 kDa) but allowed culture nutrients and most other cytokines, including chemokine C–C motif ligand (CCL) 17 and CCL22 (<20 kDa). The transwell system was incubated at 37°C for 2 h before the counting of crystal violet–positive cells on the bottom side of membranes.

### Interferon-α Stimulation

J774.2 cells were also cultured alone in dishes, followed by collection of supernatants. The concentrations of interferon-α were determined in the supernatants. Keratinocytes received 2-day stimulation of J774.2 supernatant or recombinant mouse interferon-α (PBL Assay Science, Piscataway, NJ, USA), which had identical concentrations of interferon-α (7 pg/ml) in culture media. Cell lysates were processed for extraction of total mRNA or proteins.

### Enzyme-Linked Immunosorbent Assay (ELISA)

The supernatants of the keratinocyte cultures were harvested for the detection of CCL17 and CCL22. Sandwich ELISAs were done according to the instructions for the two commercial kits (R&D Systems). The actual values were calculated from the standard curves generated by using calibrator diluents. Similarly, interferon-α in the J774.2 supernatants was determined by an ELISA kit (Elabscience Biotech, Wuhan, China).

### Statistical Analysis

All data were expressed as means ± SEM. The Stata 10.0 software package (StataCorp, College Station, TX, USA) was used for statistical analysis. Analysis of variance was used for the comparison of more than two groups. In comparing two groups, a two-tailed Student’s *t*-test was used for statistical differences. Chi-square test was used for comparing the ratios in different groups. Differences were considered significant at *p* < 0.05.

## Results

### TWEAK/Fn14 Signals Are Activated in Skin Lesions of Patients with Lupus Erythematosus

**Figure 1:**
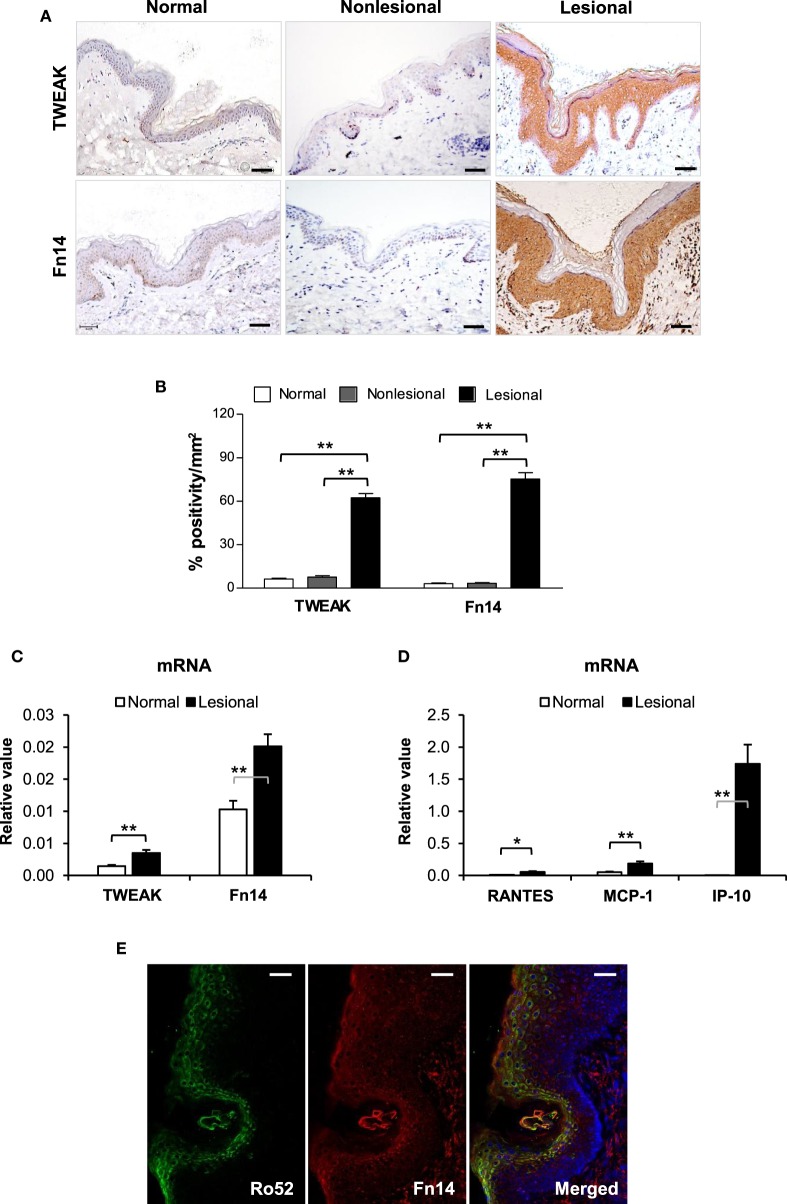
TWEAK and Fn14 expression increases in skin lesions of cutaneous lupus erythematosus. **(A)** By immunohistochemistry, the expression of TWEAK and Fn14 was determined in paraffin sections. **(B)** The stained sections were quantitated for the positivity percentage per square millimeter values. **(C)** The mRNA expression levels of TWEAK and Fn14 were determined in fresh tissues. **(D)** The mRNA levels of regulated on activation normal T cell expressed and secreted (RANTES), monocyte chemoattractant protein 1 (MCP-1), and interferon gamma-induced protein-10 (IP-10) were also determined in these tissues. **(E)** Confocal microscopy was performed for Ro52 and Fn14 expression in lesional skin. Number of normal samples = 10, number of non-lesional samples = 15, number of lesional samples = 15. Data points and error bars represent mean ± SEM. Representative images are shown. Bar = 50 µm. *p < 0.05, **p < 0.01.

By immunohistochemistry, both TWEAK and Fn14 were detected in paraffin sections of human subjects, with strong staining observed in lesional skin but not in non-lesional or normal controls (Figure 1A). Quantification of positivity percentage per square millimeter showed significantly increased expression of TWEAK and Fn14 in lesional skin (Figure 1B). There were no differences in TWEAK or Fn14 expression between non-lesional and normal controls (Figures 1A,B) or between the clinical subtypes of CLE (Figure S2 in Supplementary Material). The mRNA expression of TWEAK and Fn14 was further determined in fresh tissues, showing higher levels in lesional skin when compared with normal controls (Figure 1C). Accordingly, the mRNA expression levels of RANTES, monocyte chemoattractant protein 1 (MCP-1), and interferon gamma-induced protein 10 (IP-10) increased in lesional samples (Figure 1D). Under confocal microscopy, the Fn14 and Ro52 expression showed high overlap in upper epidermis of lesional skin (Figure 1E). Moreover, both immunohistochemistry and qRT-PCR confirmed more Ro52 and RANTES expression in tissue samples of patients with CLE (Figure S3 in Supplementary Material).

Previous studies had demonstrated that UVB irradiation increases Fn14 expression in lesional skin of MRL/lpr mice (16, 17). In this study, we determined TWEAK expression in skin lesions of MRL/lpr mice. By immunohistochemistry, TWEAK expression was stronger in MRL/lpr mice when compared with MRL/MpJ mice, and UVB irradiation even promoted TWEAK expression in MRL/lpr mice (Figure S4 in Supplementary Material).

### TWEAK Strengthens the UVB Effect on Ro52 Expression in Keratinocytes

**Figure 2:**
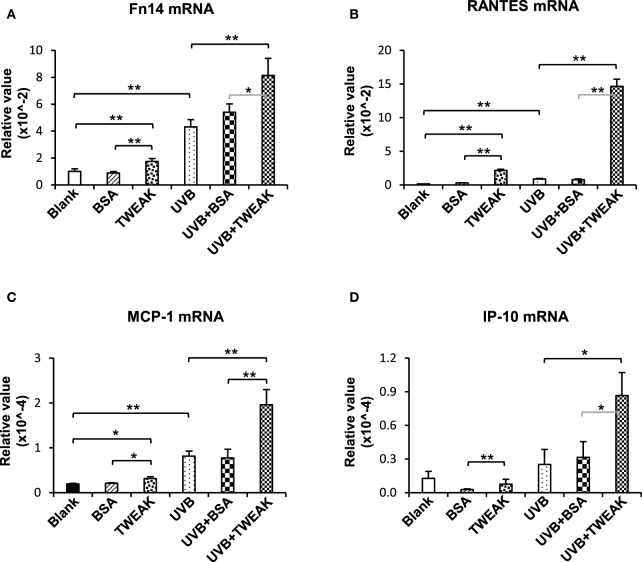
TWEAK enhances the production of proinflammatory cytokines in keratinocytes upon UVB irradiation. PAM212 keratinocytes were cultured *in vitro* and received UVB irradiation or stimulation of bovine serum albumin (BSA) or TWEAK. Quantitative reverse transcription polymerase chain reaction was performed to determine the mRNA expression levels of Fn14 **(A)**, regulated on activation normal T cell expressed and secreted (RANTES) **(B)**, monocyte chemoattractant protein 1 (MCP-1) **(C)**, and interferon gamma-induced protein 10 (IP-10) **(D)** in keratinocytes. Data were from three independent experiments. Data points and error bars represent mean ± SEM. *p < 0.05; **p < 0.01.

**Figure 3:**
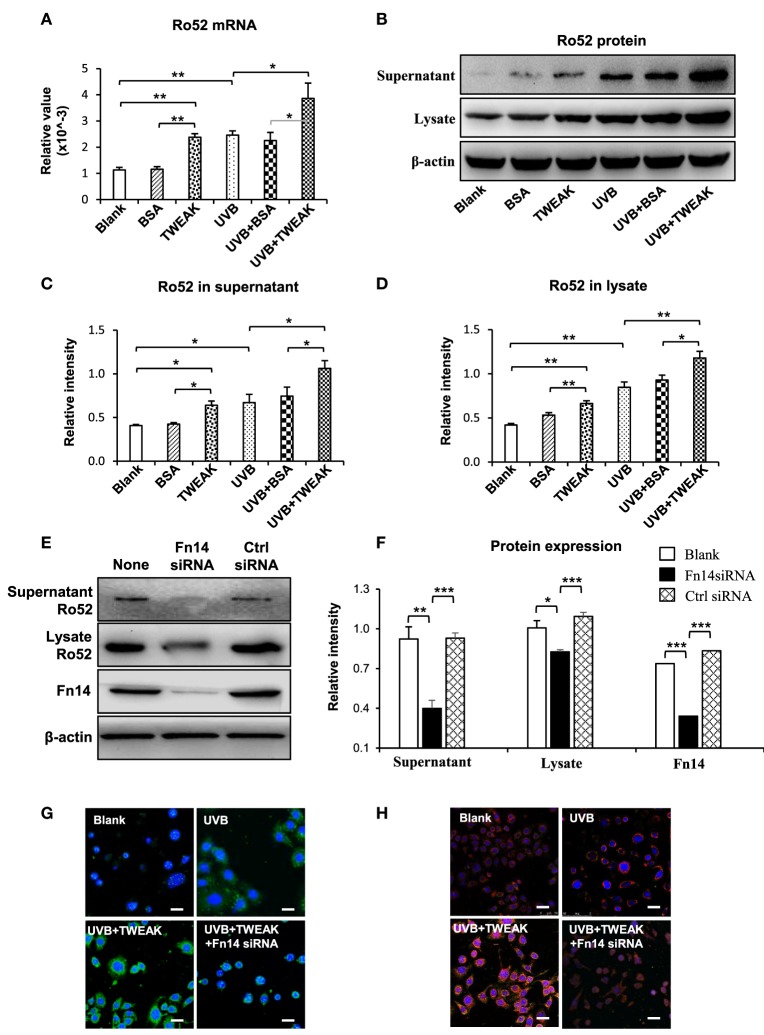
TWEAK promotes Ro52 expression in murine keratinocytes. PAM212 cells were cultured *in vitro* and received ultraviolet B (UVB) irradiation or stimulation of bovine serum albumin (BSA) or TWEAK. **(A)** The mRNA expression levels of Ro52 were determined in keratinocytes. **(B)** Western blotting was performed to detect Ro52 protein in supernatants and cell lysates. **(C,D)** The intensities of Western blotting bands were measured and then normalized to the values of β-actin. **(E,F)** By Western blotting, Ro52 protein was detected after cells were transfected with factor-inducible 14 (Fn14) or control siRNA, and then received UVB plus TWEAK treatments. The bands were measured by using ImageJ software and then normalized to the β-actin values accordingly. **(G)** By immunofluorescence, Ro52 was detected in keratinocytes that received different treatments. **(H)** Similarly, Fn14 was also detected by immunofluorescence in these cells. Data were from three independent experiments. Data points and error bars represent mean ± SEM. Representative images are shown. Bar = 5 µm. *p < 0.05, **p < 0.01, ***p < 0.001.

To show the effect of TWEAK/Fn14 activation on Ro52 expression, murine PAM212 keratinocytes were treated with UVB irradiation or plus TWEAK stimulation. We found that the mRNA levels of Fn14, RANTES, MCP-1, and IP-10 increased significantly upon UVB irradiation, which was further augmented by TWEAK (Figure 2). Moreover, TWEAK enhanced the Ro52 mRNA expression in keratinocytes, regardless of whether they received UVB irradiation or not (Figure 3A). Western blotting analysis further confirmed the increase in Ro52 levels in both the cultural supernatants and cell lysates (Figures 3B–D). To exclude the effect of signals other than those of Fn14, keratinocytes were transfected with Fn14 siRNA, followed by UVB irradiation or plus TWEAK stimulation. Western blotting showed that the enhancement effect of TWEAK on Ro52 expression was abrogated by Fn14 siRNA transfection in UVB-irradiated keratinocytes (Figures 3E,F). Furthermore, immunofluorescence verified such decrease in both Ro52 and Fn14 expression induced by Fn14 siRNA (Figures 3G,H).

To further reveal whether Ro52 participates in TWEAK-induced photosensitization, murine keratinocytes were transfected with Ro52 siRNA before TWEAK stimulation or UVB irradiation. The results showed that transfection of Ro52 siRNA significantly reduced cell apoptosis induced by TWEAK stimulation, UVB irradiation or their combination (Figure S5 in Supplementary Material).

### The NF-κB and Phosphatidylinositide 3-Kinase (PI3K)/Akt Pathways Mediate the TWEAK Promotion of Ro52 Expression

**Figure 4:**
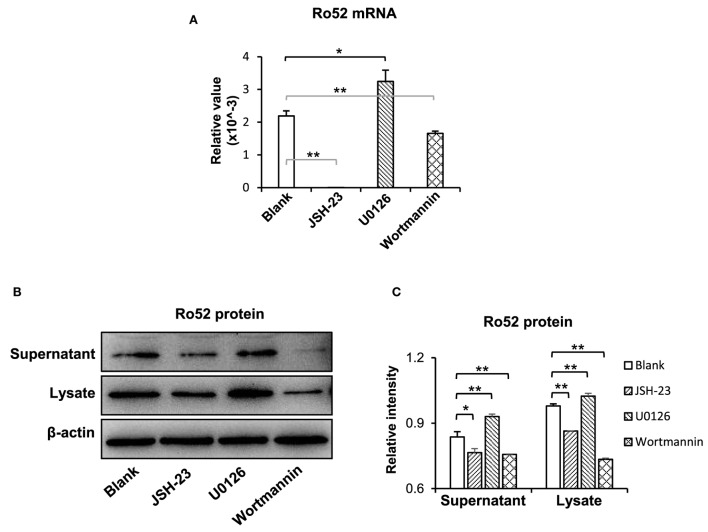
The pathway inhibitors affect the TWEAK/Fn14 regulation of Ro52 in murine keratinocytes. PAM212 cells were cultured *in vitro* and received ultraviolet B irradiation and TWEAK stimulation. Some cells were pretreated with the pathway inhibitors of nuclear factor-kappa B (JSH-23), mitogen-activated protein kinases/extracellular signal-regulated kinase (U0126), and phosphatidylinositide 3-kinase (wortmannin), respectively. **(A)** Quantitative reverse transcription polymerase chain reaction was performed for the mRNA expression levels of Ro52. **(B)** Accordingly, the protein expression levels of Ro52 were determined in supernatants and cell lysates. **(C)** The intensities of Western blotting bands were measured by using ImageJ and then normalized to the β-actin values accordingly. Data were from three independent experiments. Data points and error bars represent mean ± SEM. Representative images are shown. *p < 0.05, **p < 0.01.

**Figure 5:**
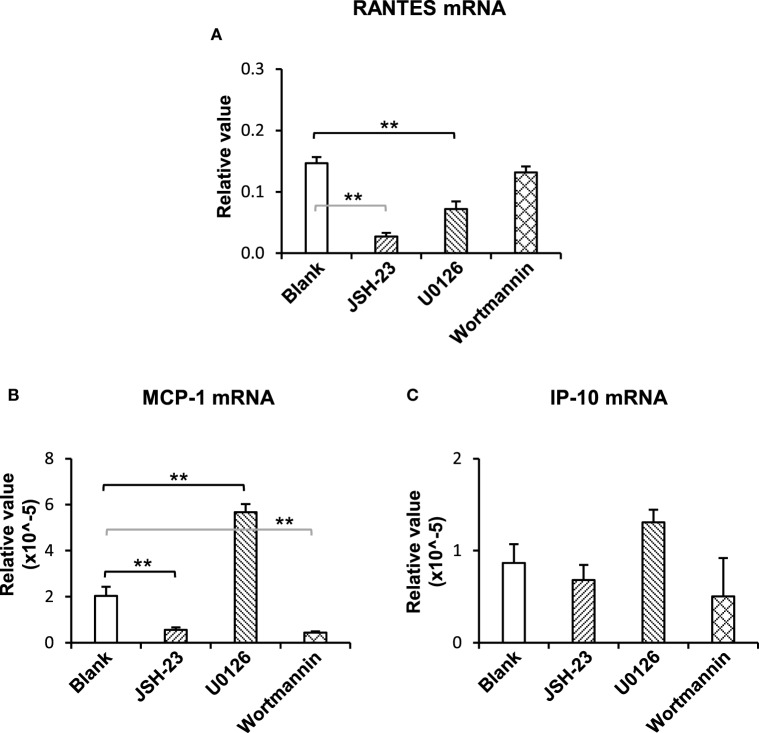
The pathway inhibitors affect the TWEAK regulation of proinflammatory cytokines in murine keratinocytes. PAM212 cells were cultured *in vitro* and received ultraviolet B irradiation and TWEAK stimulation. Some cells were pretreated with the specific inhibitors of nuclear factor-kappa B (JSH-23), mitogen-activated protein kinases/extracellular signal-regulated kinase (U0126), and phosphatidylinositide 3-kinase (wortmannin) pathways. Quantitative reverse transcription polymerase chain reaction was performed to determine the mRNA expression levels of regulated on activation normal T cell expressed and secreted (RANTES) **(A)**, monocyte chemoattractant protein 1 (MCP-1) **(B)**, and interferon gamma-inducible protein 10 (IP-10) **(C)**. Data were from three independent experiments. Data points and error bars represent mean ± SEM. **p < 0.01.

To understand the downstream signals in the TWEAK/Fn14 regulation of Ro52 expression, keratinocytes were pretreated with specific inhibitors of the NF-κB (JSH-23), mitogen-activated protein kinases (MAPK)/ERK (U0126), and PI3K/Akt (wortmannin) pathways, respectively. Then, these cells received UVB plus TWEAK treatments. The results showed that JSH-23 and wortmannin decreased the Ro52 mRNA levels, whereas U0126 promoted Ro52 expression (Figure 4A). Accordingly, Western blotting analysis showed similar trends in keratinocytes pretreated with these inhibitors (Figures 4B,C). The expression levels of NF-κB, ERK, and Akt proteins were also determined, showing that these specific inhibitors significantly decreased their expression accordingly (Figure S6 in Supplementary Material). To further confirm the effect of these inhibitors, the mRNA levels of RANTES, MCP-1, and IP-10 were determined in keratinocytes (Figure 5). JSH-23 was found to decrease the production of RANTES and MCP-1. U0126 and wortmannin had different effects on the two cytokines, whereas IP-10 was not affected by any of the three inhibitors.

### TWEAK/Fn14 Activation in Keratinocytes Induces Macrophage Chemoattraction

**Figure 6:**
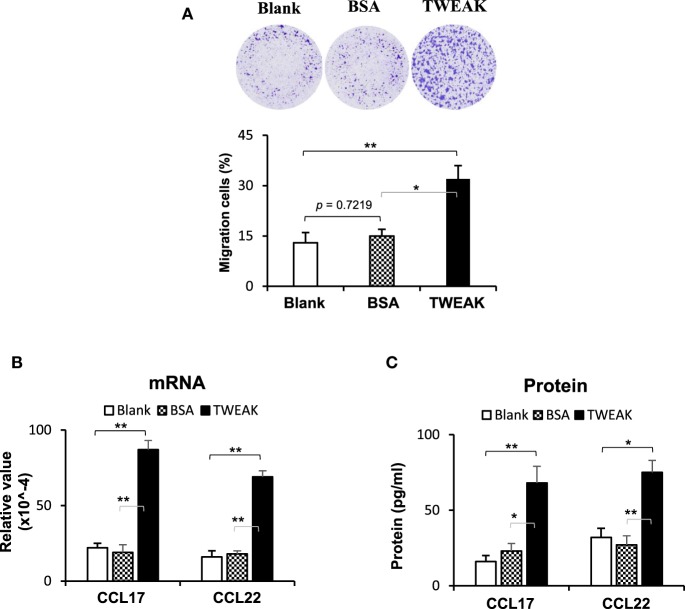
TWEAK/Fn14 activation in keratinocytes induces macrophage chemoattraction. In a designed transwell system, murine PAM212 keratinocytes in an outer insert were stimulated with TWEAK or bovine serum albumin (BSA). Murine J774.2 macrophages cultured in an inner insert were observed for migration. The migration amount was determined based on crystal violet-stained cells as a percentage of the total cells. **(A)** The percentages of migrated macrophages were assessed. **(B)** The mRNA expression levels of CCL17 and CCL22 were determined in TWEAK-stimulated keratinocytes. **(C)** By enzyme-linked immunosorbent assay, the CCL17 and CCL22 proteins were measured in the supernatants of TWEAK-stimulated keratinocytes. Data were from three independent experiments. Data points and error bars represent mean ± SEM. Representative images are shown. *p < 0.05, **p < 0.01.

Dermal infiltration of macrophages contributes to the development of CLE through the production of interferon-α (2), which may induce the upregulation and nuclear translocation of Ro52 in cells (22). Hence, we studied the effect of TWEAK/Fn14 activation on macrophage chemotaxis. The results showed that the addition of TWEAK to keratinocyte cultures (not contacting macrophages) enhanced macrophage migration (Figure 6A). Moreover, compared with blank and BSA groups, these keratinocytes expressed more CCL17 and CCL22 on TWEAK stimulation (Figures 6B,C).

### Interferon-α Enhances Ro52 Expression in Keratinocytes

The effect of macrophage-derived interferon-α on Ro52 expression in keratinocytes was also analyzed. The results showed that the supernatants of J774.2 cells had high levels of interferon-α (4.65–9.53 ng/ml). The keratinocytes stimulated with J774.2 supernatant or recombinant interferon-α expressed more Ro52 at mRNA and protein levels (Figure S7 in Supplementary Material).

### TNFR and TRAF2 Are Pivotal in the TWEAK Effect on Keratinocytes

**Figure 7:**
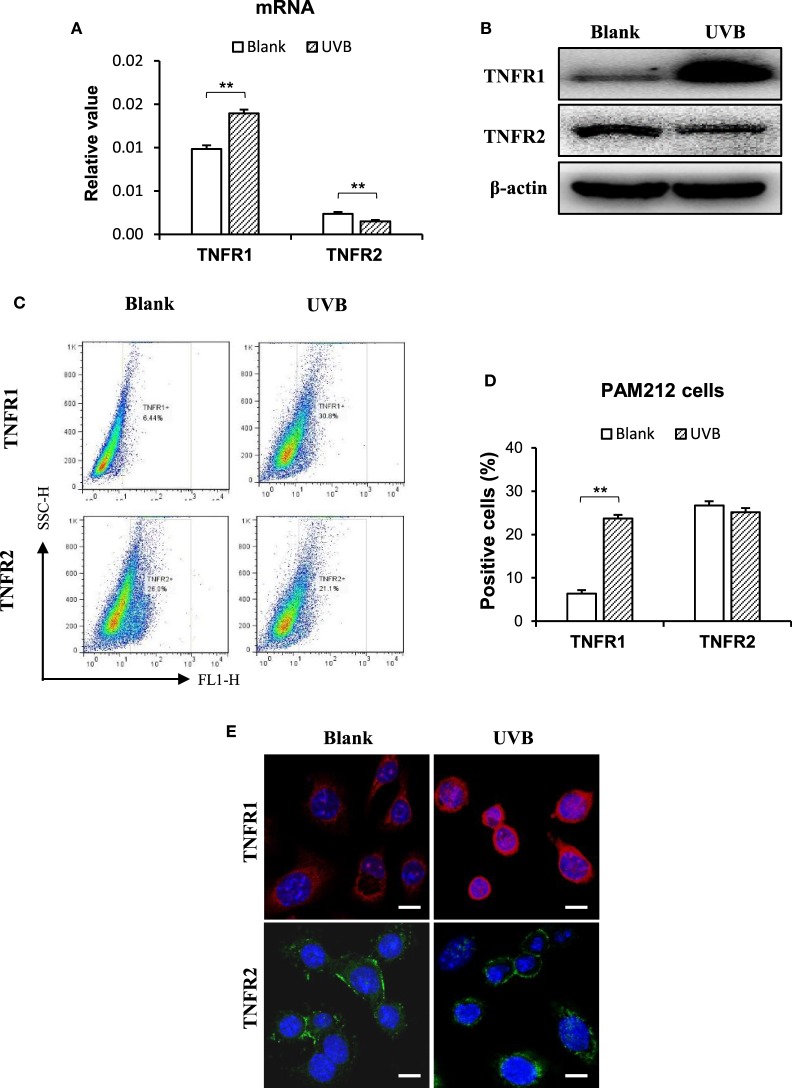
Ultraviolet B (UVB) irradiation enhances TNF receptor type 1 (TNFR1) expression in murine keratinocytes. PAM212 cells were cultured *in vitro* and received UVB irradiation. **(A)** The mRNA expression levels of TNFR1 and TNFR2 in keratinocytes were quantitated by quantitative reverse transcription polymerase chain reaction. **(B)** Western blotting was performed for the TNFR1 and TNFR2 proteins in cell lysates. **(C,D)** The expression of TNFR1 and TNFR2 in keratinocytes was determined by flow cytometry. **(E)** TNFR1 and TNFR2 expression was detected in keratinocytes by immunofluorescence. Data were from three independent experiments. Data points and error bars represent mean ± SEM. Representative images are shown. **p < 0.01.

The effect of TWEAK on the regulation of cell fate depends on TNFR (12, 23). Thus, the expression of TNFR subtypes was determined in keratinocytes irradiated with UVB. Interestingly, UVB irradiation promoted the mRNA expression of TNFR1 but inhibits that of TNFR2 in these cells (Figure 7A). Western blotting confirmed such effect of UVB irradiation on TNFR subtypes in cell lysates (Figure 7B). Moreover, both flow cytometry (Figures 7C,D) and immunofluorescence (Figure 7E) verified the shift in TNFR expression profile in these cells.

**Figure 8:**
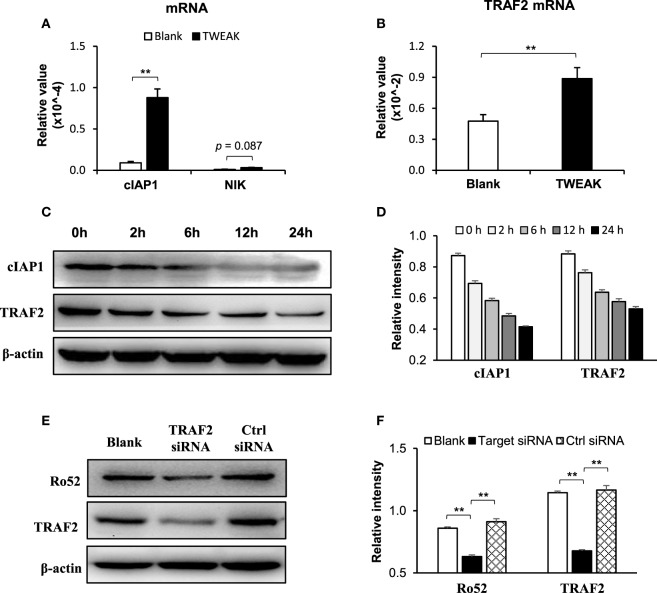
TNF receptor associated factor 2 (TRAF2) mediates the TWEAK-induced Ro52 upregulation in murine keratinocytes. PAM212 cells were cultured *in vitro* and received both ultraviolet B irradiation and TWEAK stimulation. **(A)** The mRNA expression levels of cellular inhibitor of apoptosis protein 1 (cIAP1) and NF-κB-inducing kinase (NIK) were determined by quantitative reverse transcription polymerase chain reaction. **(B)** Similarly, the mRNA expression levels of TRAF2 were measured in these cells. **(C,D)** Western blotting was performed for cIAP1 and TRAF2 proteins at different time points after the addition of TWEAK. **(E,F)** By Western blotting, Ro52 protein was detected in keratinocytes transfected with TRAF2 or control siRNA. Data were from three independent experiments. Data points and error bars represent mean ± SEM. Representative images are shown. **p < 0.01.

In these keratinocytes, TWEAK increased the mRNA levels of cIAP1 and TRAF2 but showed no effect on the mRNA expression of NF-κB-inducing kinase (NIK) (Figures 8A,B). Moreover, TWEAK reduced the protein expression of cIAP1 and TRAF2 in a time-dependent manner (0–24 h) (Figures 8C,D). Furthermore, the transfection of TRAF2, but not of control siRNA, decreased the Ro52 protein level in keratinocytes treated with UVB irradiation plus TWEAK stimulation (Figures 8E,F).

## Discussion

In this study, we showed that TWEAK/Fn14 activation is prominent in skin lesions of patients with CLE. Exogenous TWEAK not only strengthens the UVB enhancement effect on the expression of Ro52 and proinflammatory cytokines in keratinocytes through the NF-κB and PI3K/Akt signals but also significantly favors *in vitro* chemoattraction of macrophages. Moreover, Ro52 inhibition attenuates apoptosis of keratinocytes induced by TWEAK stimulation. Interestingly, UVB irradiation induces the predominance of TNFR1 over TNFR2 expression in keratinocytes. Furthermore, the inhibition of Fn14 or TRAF2 abrogates the enhancement effect of UVB irradiation on Ro52 expression. Therefore, TWEAK/Fn14 activation exacerbates Ro52-mediated photosensitization in keratinocytes and involves the function of TNFR and TRAF2 partners.

Previously, TWEAK and Fn14 overexpression was found in several skin inflammatory diseases including atopic dermatitis, psoriasis, and even human papillomavirus infection (12, 23, 24). However, TWEAK/Fn14 activation leads to different fates (death or proliferation) of keratinocytes under these inflammations. Recently, we found that Fn14 is highly expressed in skin lesions of both MRL/lpr mice and patients with CLE (16). UVB irradiation promotes Fn14 expression in keratinocytes and increases RANTES expression and keratinocyte apoptosis on addition of TWEAK (16). Moreover, Fn14 deficiency protects MRL/lpr mice from histologic lupus erythematosus-like skin inflammation induced by UVB light (17). Our results are consistent with these previous findings and even provide more evidence that the TWEAK/Fn14 pathway is activated in CLE. We found that TWEAK and downstream proinflammatory cytokines, including RANTES, MCP-1, and IP-10, are also highly expressed in skin lesions. Moreover, Ro52 expression increases in lesional tissues accordingly and overlaps with Fn14 expression in the epidermis of lesional skin. The slight discrepancy of staining pattern between the immunohistochemical and immunofluorescent results might be due to the differences in antibodies and antigen exposure. Hence, the TWEAK/Fn14 signals are activated in CLE and correlates with Ro52 upregulation.

Ultraviolet B, rather than UVA, is the main source that induces cell surface expression and relocation of Ro52 (25). The Ro52 overexpression and photosensitization are central in the pathogenesis of CLE (7, 8). Recent studies showed that UVB irradiation upregulates both Fn14 and Ro52 in cutaneous keratinocytes (8, 16), the latter of which further increases the production of cytokines, including type 1 interferon (9). In this study, Ro52 is highly expressed in skin lesions of CLE that are characterized by overexpression of both TWEAK and Fn14. Moreover, TWEAK alone upregulates Ro52 in cultured keratinocytes and also enhances the UVB effect on Ro52 expression in cells. Meanwhile, Fn14 siRNA transfection can abrogate the promotion effect of TWEAK on Ro52 expression. Therefore, TWEAK/Fn14 interaction amplifies UVB-induced Ro52 expression in keratinocytes. In fact, Ro52 regulates the production of type 1 interferon and other cytokines in cells (9). TWEAK also upregulates proinflammatory cytokines, such as RANTES, MCP-1, and IP-10, which may be induced by interferon γ in keratinocytes (26–28). Although TWEAK and UVB cooperate in the upregulation of Ro52 in keratinocytes, they may induce the lesions of CLE in separate pathways. UVB enhances Ro52 expression and cell surface relocation by oxidative stress signals and causes DNA damage or gene mutation directly (25, 29). We found that TWEAK acts on keratinocytes mainly through proinflammatory signaling pathways. These two factors mutually contribute to Ro52-mediated photosensitization in CLE.

It is known that TWEAK regulates junctional proteins in cells *via* canonical NF-κB pathway and ERK activation (30). Our results showed that the TWEAK-induced Ro52 expression in keratinocytes can be suppressed by NF-κB or PI3K/Akt inhibitor but enhanced by MAPK/ERK inhibitor, suggesting that the NF-κB and PI3K pathways participate in the TWEAK regulation of Ro52. Actually, Ro52 expression is mainly induced by interferon-α, while NF-κB activation contributes to the gene transcription of interferon-α (31). Expression of NF-κB p50 subunit can promote the activity of *Ifnb* promoter to upregulate the expression of interferon-α (32), which further enhances the expression of Ro52 (22). Consistently, our results showed Ro52 expression increases in keratinocytes after stimulation of interferon-α or macrophage supernatant that contains high level of interferon-α. Moreover, PI3K/Akt signals may also activate the NF-κB pathway, resulting in enhanced expression of downstream molecules (33). Therefore, both PI3K/Akt and NF-κB pathways mediate TWEAK upregulation of Ro52. We also found that MAPK/ERK inhibition correlates with increased Ro52 expression. The MAPK/ERK pathway can function independently in cells, and its activation is usually associated with cell proliferation and survival (34). Since Ro52 is preferably expressed in cells undergoing death and promotes apoptosis by regulating Bcl-2 production (35), MAPK/ERK activation may reversely inhibit Ro52 expression and apoptosis. Obviously, TWEAK/Fn14 interaction upregulates Ro52 expression through multiple signaling pathways that may act synergistically or independently.

**Figure 9:**
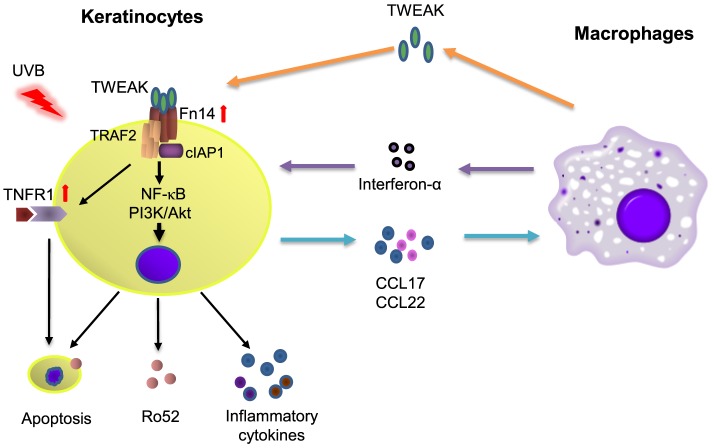
The diagram for TWEAK/factor-inducible 14 (Fn14) regulation of Ro52 expression and other downstream signals. Ultraviolet B (UVB) irradiation enhances the expression of both Fn14 and TNF receptor type 1 (TNFR1) in keratinocytes. The binding of TWEAK to Fn14 initiates the formation of Fn14/TNF receptor associated factor 2 (TRAF2)/cellular inhibitor of apoptosis protein 1 (cIAP1) complex, which further interacts with the TNFR1 partner and triggers cell apoptosis as well as surface expression of Ro52. Fn14/TRAF2/cIAP1 complex can also activate the nuclear factor-kappa B (NF-κB) and phosphatidylinositide 3-kinase (PI3K)/Akt pathways to upregulate the expression of Ro52 and inflammatory cytokines, the latter of which recruit macrophages. Infiltrating macrophages secrete proinflammatory cytokines including TWEAK and interferon-α, which together aggravate immune responses in cutaneous lupus erythematosus.

Macrophage infiltration is prominent in skin lesions of CLE and contributes to the dermal production of interferon-α (2), which can upregulate Ro52 in HeLa cells (22). Consistently, we found that TWEAK stimulation of cultured keratinocytes induces chemotaxis of macrophages. In fact, Fn14 deficiency in MRL/lpr mice was related with a decrease in Iba-1 (macrophage marker) positive cells in skin lesions (16). Depletion of macrophages can be protective against the development of skin lesions after UVB exposure (17). Moreover, our results showed that keratinocytes express more CCL17 and CCL22 on TWEAK stimulation. CCL17 and CCL21 can be secreted by keratinocytes (36) and also contribute to the local recruitment of macrophages (37). The expression levels of CCL17 and CCL22 correlates with CCL19 in inflamed lungs, which further leads to the accumulation of macrophages (38). Additionally, TWEAK induces MCP-1 in UVB-irradiated keratinocytes, which is also a chemoattractant of macrophages (39). Therefore, the TWEAK/Fn14 activation-induced macrophage chemotaxis may facilitate Ro52 expression and inflammatory responses in CLE (Figure 9).

TNF receptor associated factor 2 is a TNFR-associated protein and mediates the signal transduction from members of the TNFR superfamily (40). Fn14 activation triggers the assembly of cIAP1, TRAF2, and other components (41). Transfection of human papillomavirus E6/E7 genes can enhance the expression of TRAF2, TNFR2, and Fn14 in keratinocytes (12). Therefore, the Fn14, TRAF2, and TNFR partners may participate in the function of TWEAK in cells through direct interaction or indirect signal transduction. In this study, UVB-irradiated keratinocytes express higher mRNA levels but lower protein levels of cIAP1 and TRAF2 on TWEAK stimulation. This phenomenon is in accordance with the facts that TWEAK promotes the recruitment of cIAP1 and TRAF2 to the Fn14 signaling complex leading to cIAP1 autoubiquitination and degradation (41). The higher levels of mRNA expression may be due to a feedback manner. TWEAK has no effect on the mRNA expression of NIK, indicating that TRAF2 promotion does not occur through the non-canonical NF-κB pathway. Moreover, the transfection of TRAF2 siRNA can reduce Ro52 protein in keratinocytes that receive UVB irradiation and TWEAK stimulation. Hence, the Fn14-TRAF2 signals are instrumental in the TWEAK regulation of Ro52. In contrast to TNFR1, which induces cell death through a death domain, TNFR2 activation triggers antiapoptotic reactions (40). Our results showed that UVB irradiation promotes the expression of TNFR1 but inhibits that of TNFR2 in keratinocytes. This explains the phenomenon that TWEAK/Fn14 activation induces keratinocyte death under UVB irradiation. In fact, the biased expression of TNFR1 and TNFR2 is observed in keratinocytes under different inflammation conditions, such as HPV infection and psoriasis, causing different cell fates on TWEAK stimulation (12, 23). Moreover, intracellular Ro52 in keratinocytes can be upregulated by activating TNFR1 but not TNFR2 (42), although which both associate with TRAF2 during signal transduction (40). Therefore, an Fn14-TRAF2-TNFR axis may function in the TWEAK/Fn14-induced Ro52 expression and other downstream signals (Figure 9). However, the precise mechanism remains to be elucidated.

In summary, TWEAK/Fn14 signals are activated in CLE and directly enhance the UVB effect on Ro52 expression in keratinocytes. Besides, TWEAK/Fn14 activation in keratinocytes induces macrophage chemoattraction, which may upregulate Ro52 in cells indirectly. Furthermore, UVB irradiation preferably induces a TNFR1 expression profile in keratinocytes. The NF-κB and PI3K/Akt pathways as well as the TRAF2/TNFR partners are involved in the TWEAK-upregulated Ro52 expression. Targeting the TWEAK/Fn14 pathway may be a novel strategy in the management of Ro52-mediated photosensitization in CLE.

## Ethics Statement

This study was carried out in accordance with the recommendations of the guidelines of the Hospital Research Ethics Committee with written informed consent from all subjects. All subjects gave written informed consent in accordance with the Declaration of Helsinki. The protocol was approved by the Hospital Research Ethics Committee.

## Author Contributions

YL participated in the design of the study, and performed most experimental work. MX, XM, KW, and TZ carried out some experiments. KL and SX discussed the experimental data and contributed to the interpretation of results. YX conceived and designed the study and prepared the manuscript. All the authors read and approved the final manuscript.

## Conflict of Interest Statement

The authors declare that the research was conducted in the absence of any commercial or financial relationships that could be construed as a potential conflict of interest.
