# Supplementary material for: TWEAK/Fn14 Activation Participates in Ro52-Mediated Photosensitization in Cutaneous Lupus Erythematosus
Source: Front Immunol. 2017 May 31;8:651. doi: 10.3389/fimmu.2017.00651 (PMC5449764; doi:10.3389/fimmu.2017.00651)
Supplement: Supplementary file 1 [file Image_1.PDF]

Supplementary Table S1. Demographic characteristics of CLE patients and healthy donors

No.	Sex (F/M)	Age (y)	Course (y/m/d)	Biopsy Site	Diagnosis	No.	Sex (F/M)	Age (y)
1[1,2,3]	M	39	1 y	Face	DLE	H 1[1,2]	F	72
2[1,2,3]	M	46	3 y	Scalp	DLE	H 2[1,2]	F	73
3[1,2,3]	M	60	6 m	Face	DLE	H 3[1,2]	M	55
4[1,2,3]	F	31	4 y	Scalp	DLE	H 4[1,2]	F	64
5[1,2,3]	F	65	6 m	Underlip	DLE	H 5[1,2]	M	75
6[1,2,3]	M	21	15 d	Neck	SCLE	H 6[1,2]	M	69
7[1,2,3]	M	25	1 m	Trunk	SCLE	H 7[1,2]	F	65
8[1,2,3]	F	30	1 y	Opisthenar	SCLE	H 8[1,2]	F	76
9[1,2,3]	F	34	7 d	Face	SCLE	H 9[1,2]	F	73
10[1,2,3]	F	25	1 y	Trunk	SCLE	H 10[1,2]	M	68
11[1,2,3]	F	32	4 y	Face	SLE			
12[1,2,3]	F	71	1 y	Opisthenar	SLE			
13[1,2,3]	F	28	2 m	Trunk	SLE			
14[1,2,3]	F	26	7 d	Face	SLE			
15[1,2,3]	M	39	1 y	Neck	SLE			

[1]Patients and healthy controls whose tissues were used for paraffin sections to detect tumor necrosis factor-like weak inducer of apoptosis (TWEAK), fibroblast growth factor-inducible 14 (Fn14), and Ro52.
[2]Patients and healthy controls whose peripheral blood mononuclear cell samples were analyzed for the mRNA levels of TWEAK, Fn14, Ro52, and other cytokines.
[3]Patients whose skin samples were also used for examining Fn14 and Ro52 expression by immunofluorescence.

Abbreviations: F, female; M, male; H, healthy; y, year; m, month; d, day

Supplementary Table S2. Primers for sequencing different expression genes

Gene	Primer (5’-3’)	Gene	Primer (5’-3’)
Human Fn14	F: CTCTGAGCCTGACCTTCGTG<br>R: GGGGGCACATTGTCACTGGA	Mouse Fn14	F: CGCTCTTAGTCTGGTCCTG<br>R: TGGATCAGTGCCACACCTG
Human GAPDH	F: GCACCGTCAAGGCTGAGAAC<br>R: TGGTGAAGACGCCAGTGGA	Mouse GAPDH	F: GCTGAGTATGTCGTGGAGT<br>R: GTTCACACCCATCACAAAC
Human IP-10	F: AGGGTGAGAAGAGATGTCTG<br>R: ATGTAGGGAAGTGATGGGAG	Mouse IP-10	F: GTGCTGCGATGGATGGACAG<br>R: GGCTGGTCACCTTTCAGAAG
Human MCP-1	F: TGTGCCTGCTGCTCATAG<br>R: TCTTTGGGACACTTGCTG	Mouse pan-IFN-α	F: CCTGAGAGAGAAGAAACACAGC<br>R: GAGGAAGACAGGGCTCTCC
Human RANTES	F: AGCCCTCGCTGTCATCCT<br>R: TCCTTGATGTGGGCACGG	Mouse MCP-1	F: GCTGGGGGAAAACTGGAGAG<br>R: CCAGCACACAGAAGAGGTCC
Human Ro52	F: GCACGCTTGACAATGATG<br>R: CCTTTCCCAACCTGAGAG	Mouse NIK	F: GACCAGAACTCCACAAACTGAT<br>R: GGGTAGAATGGGAAAGGATG
Human TWEAK	F: CCAGATCGGGGAGTTTAT<br>R: CACCATCCACCAGCAAGT	Mouse Ro52	F: AAACAGATAGCCCAGAATAC<br>R: CTTAGCAGCCTCTTCAATAG
Mouse CCL17	F: AGTGGAGTGTTCCAGGGATG<br>R: GGTCACAGGCCGTTTTATGT	Mouse TNFR1	F: GTAAGGAGACTCAGAACACCG<br>R: GGAGGTAGGCACAACTTCATA
Mouse CCL22	F: CTCCCCTCTATCCCTTCCAG<br>R: CCTATGGGCCTTCCCTACAT	Mouse TNFR2	F: GCACTAAACAGCAGAACCGA<br>R: TGATGTGGTGTCAGAGAACG
Mouse cIAP1	F: GGAGTTCCTGTGTCAGAAAGG<br>R: AGACTGTTGGCTGGATTCAA	Mouse TRAF2	F: GAGAACATTGTCTGCGTCTTG<br>R: GGATACTTCCAACTCGGAGA

Abbreviations: F: forward; R: reverse

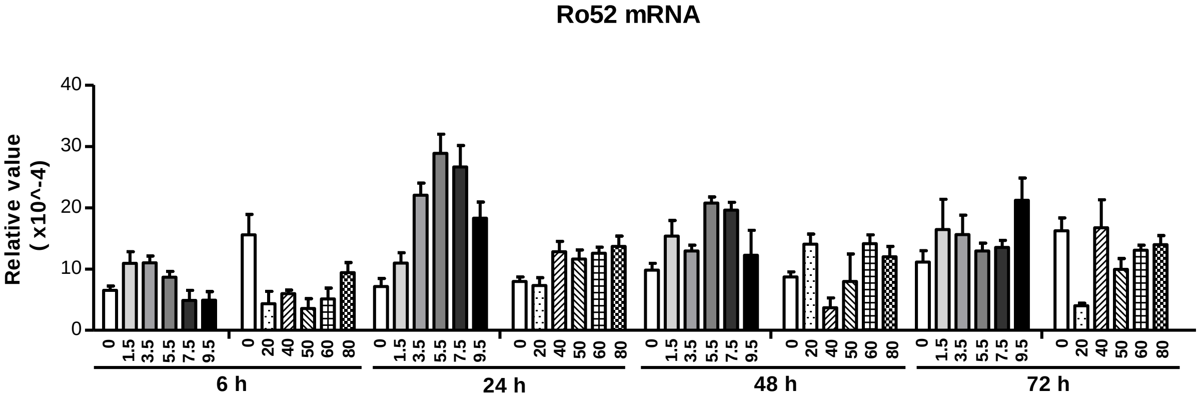

Supplementary Figure S1. The effect of UVB on the mRNA expression levels of Ro52 in keratinocytes. PAM212 cells were cultured *in vitro* and received UVB irradiation with different doses (0 to 80 mJ/cm^2) or different culture time (after irradiation). The mRNA expression levels of Ro52 were quantitated by qRT-PCR. Data were from three independent experiments. Data points and error bars represent mean $\pm$ SEM.

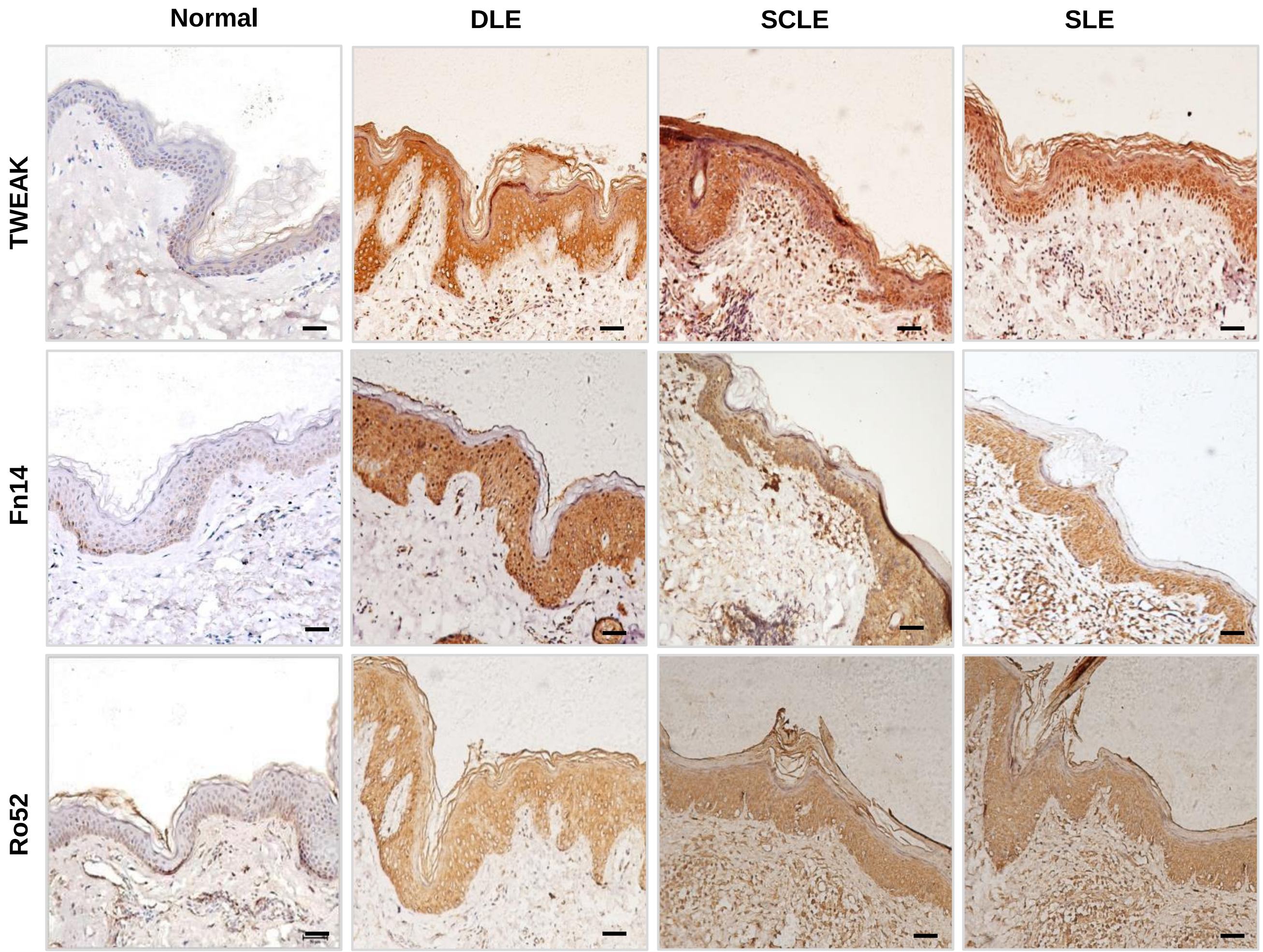

Supplementary Figure S2. The expression of TWEAK, Fn14 and Ro52 in normal or lesional skin. Immunohistochemistry was performed with paraffin sections of human subjects. The expression of TWEAK, Fn14 and Ro52 was detected in normal skin (n = 10) and lesional skin from patients with DLE (n = 5), SCLE (n = 5), or SLE (n = 5). Representative images are shown. Bar = 50 mm.

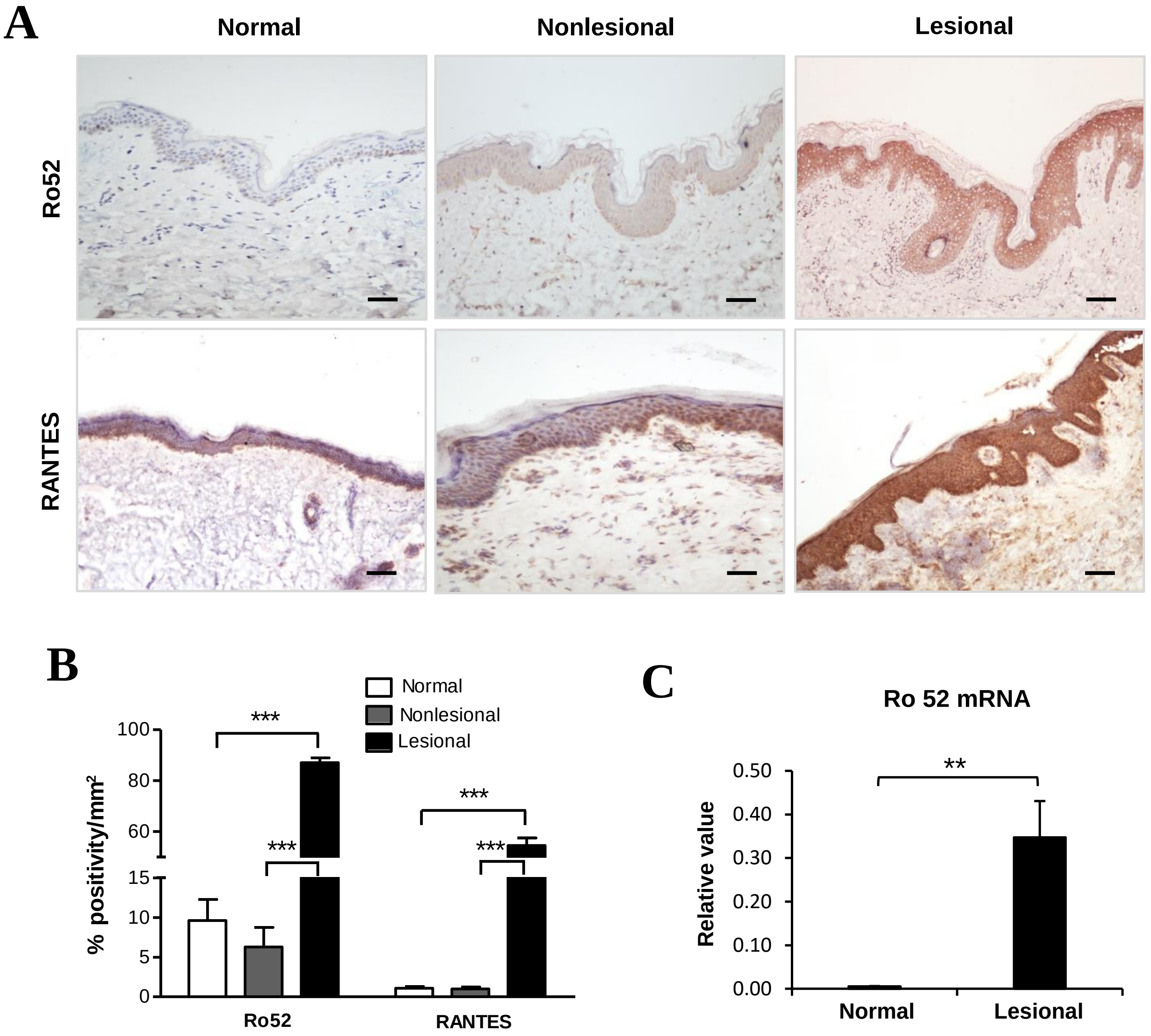

Supplementary Figure S3. The Ro52 and RANTES expression increases in the lesions of cutaneous lupus erythematosus. (**A**) By immunohistochemistry, the Ro52 and RANTES expression was detected with paraffin sections of normal, nonlesional, and lesional skin samples. (**B**) The stained sections were quantitated for the positivity percentage/mm^2 values. (**C**) The mRNA levels of Ro52 were determined in fresh tissues of normal or lesional skin. Number of normal samples = 10, of lesional (or nonlesional) samples = 15. Data points and error bars represent mean ± SEM. Representative images are shown. Bar = 50 mm. **$p < 0.01$, ***$p < 0.001$.

A

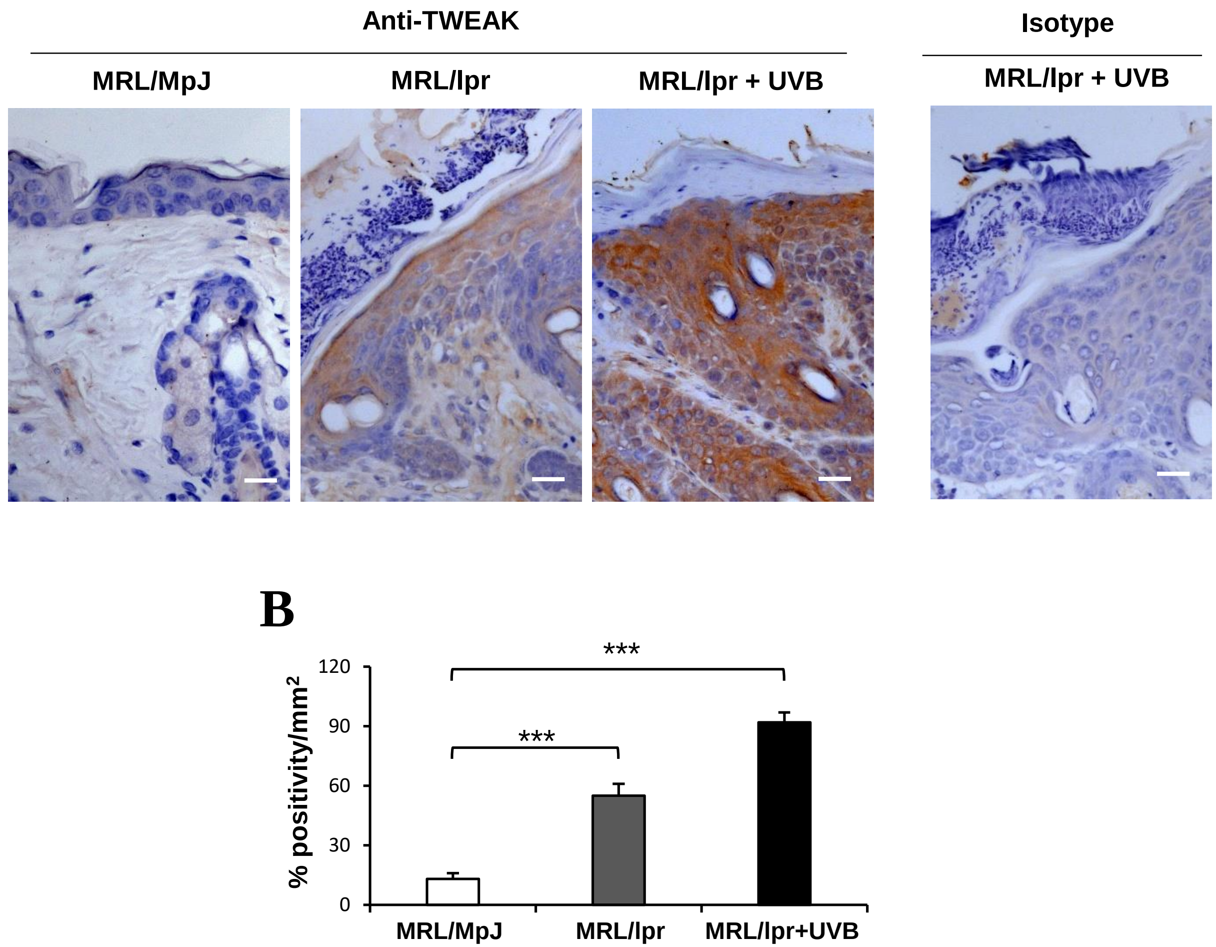

Supplementary Figure S4. TWEAK expression increases in the skin lesions of MRL/lpr mice upon UVB irradiation. (**A**) By immunohistochemistry, TWEAK expression was detected with paraffin sections of MRL/MpJ or MRL/lpr mice. (**B**) The stained sections were quantitated for the positivity percentage/mm^2 values. There were 5 mice in each group. Data points and error bars represent mean ± SEM. Representative images are shown. Bar = 100 mm. ***p < 0.001.

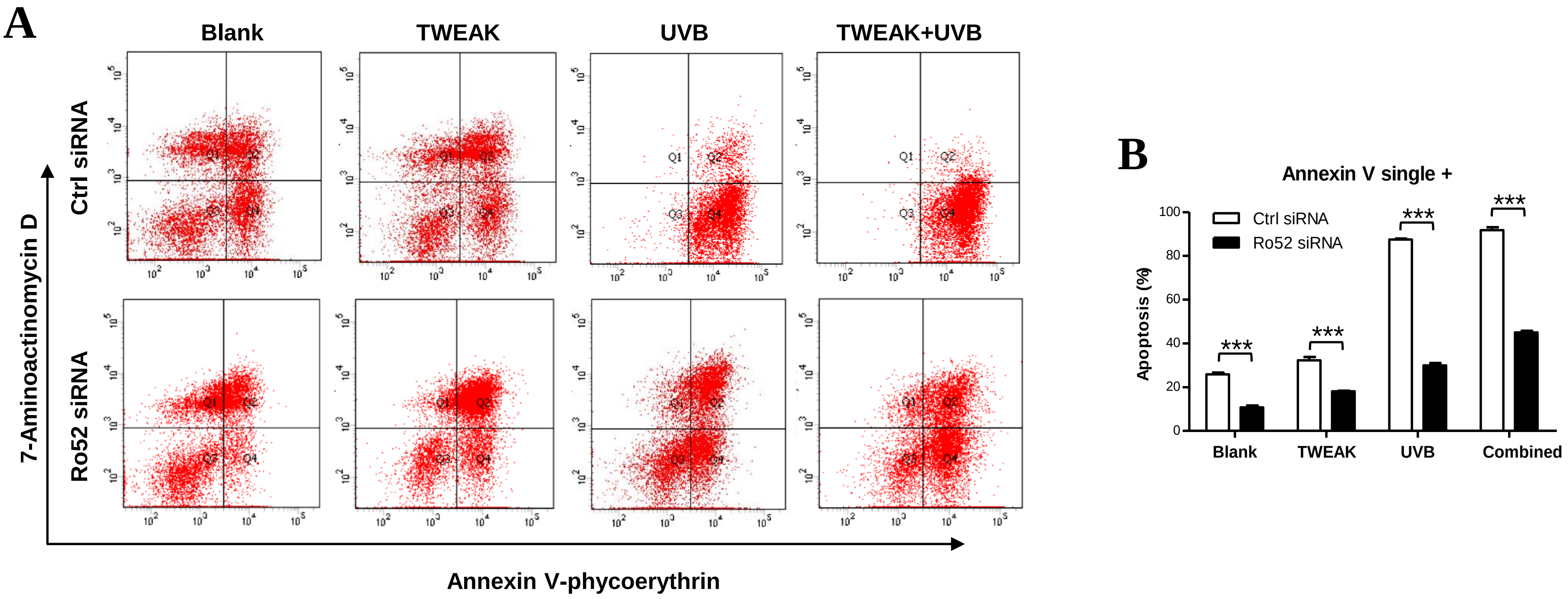

Supplementary Figure S5. Ro52 siRNA transfection abrogates keratinocyte apoptosis induced by TWEAK or UVB irradiation. PAM212 keratinocytes were transfected with control or Ro52 siRNA, followed by TWEAK stimulation, UVB irradiation or their combination. Then, cells were analyzed by flow cytometry. (**A**) Representative plots are shown. (**B**) The percentages of early apoptotic cells (annexin V single positive) in different groups. Data were from three independent experiments. Data points and error bars represent mean ± SEM. ****p* < 0.001.

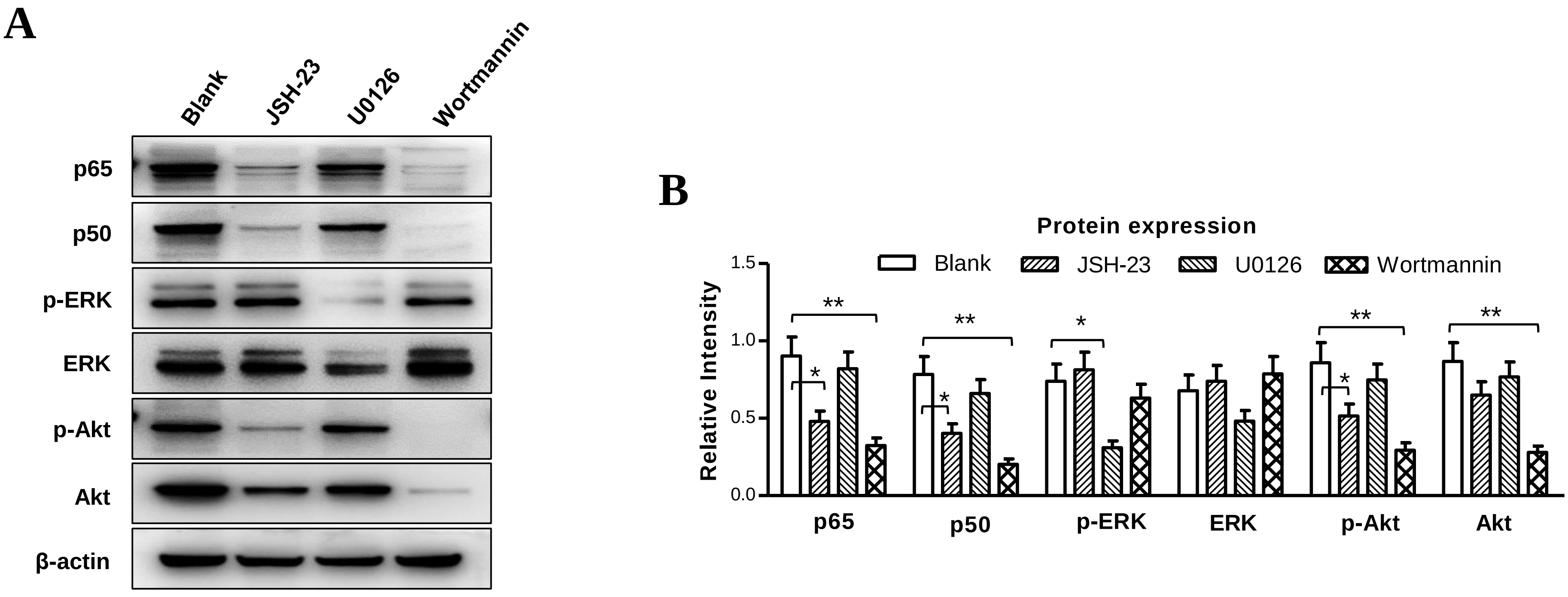

Supplementary Figure S6. The pathway inhibitors affect the TWEAK/Fn14 regulation of NF-kB, ERK and AKT proteins in murine keratinocytes. PAM212 cells were cultured *in vitro* and treated with UVB irradiation and TWEAK. Some cells were pretreated with the pathway inhibitors of NF-κB (JSH-23), MAPK/ERK (U0126), and PI3K (wortmannin), respectively. (**A**) By Western blotting, the expression of p50, p65, ERK and Akt proteins was determined. (**B**) The band intensities were measured by using ImageJ software and then normalized to the values of β-actin accordingly. Data were from three independent experiments. Data points and error bars represent mean ± SEM. Representative images are shown. *$p < 0.05$, **$p < 0.01$.

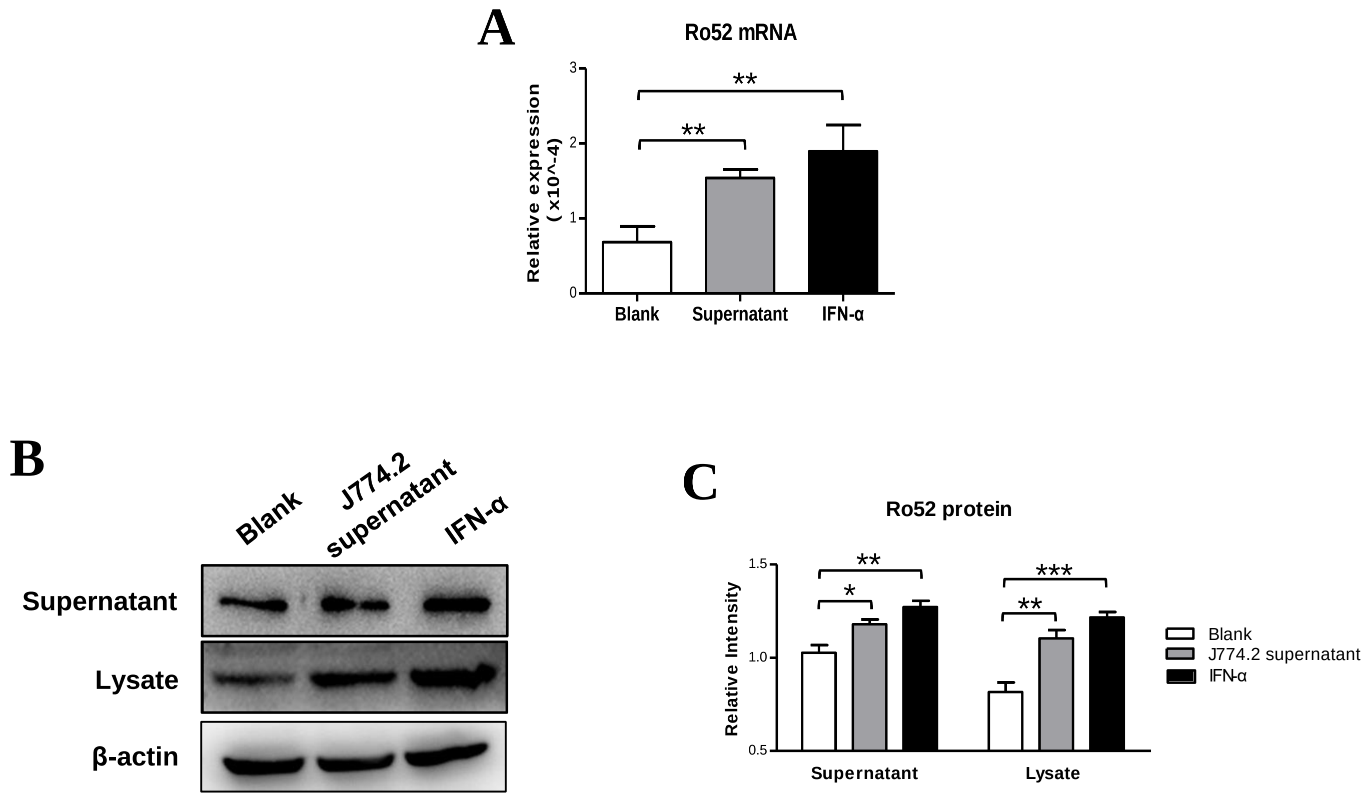

Supplementary Figure S7. The expression levels of Ro52 in keratinocytes stimulated by J774.2 supernatants or interferon (IFN)-α. PAM212 cells were cultured *in vitro* and received 2-day stimulation of J774.2 supernatants or recombinant IFN-α, which had identical concentration of IFN-α (7 pg/ml) in media. (**A**) The mRNA expression levels of Ro52 were determined by qRT-PCR. (**B**) Western blotting was performed for Ro52 protein in cell lysates or supernatants of keratinocytes. (**C**) The intensities of Western blotting bands were measured by using ImageJ software and then normalized to the values of β-actin accordingly. Data were from three independent experiments. Data points and error bars represent mean ± SEM. Representative images are shown. *$p < 0.05$, **$p < 0.01$, ***$p < 0.001$.
